# Mortality after surgery for benign prostate hyperplasia: a nationwide cohort study

**DOI:** 10.1007/s00345-022-03999-0

**Published:** 2022-04-16

**Authors:** Alisa Salmivalli, Otto Ettala, Peter J. Boström, Ville Kytö

**Affiliations:** 1 Doctoral Programme in Clinical Research, University of Turku and Turku University Hospital, Kiinamyllynkatu 4-8, 20520 Turku, Finland; 2 Department of Urology, Turku University and Turku University Hospital, Turku, Finland; 3 Heart Center, Turku University Hospital and University of Turku, Turku, Finland; 4 Research Center of Applied and Preventive Cardiovascular Medicine, University of Turku, Turku, Finland; 5 Center for Population Health Research, Turku University Hospital and University of Turku, Turku, Finland; 6 Administrative Center, Hospital District of Southwest Finland, Turku, Finland; 7 Department of Public Health, University of Helsinki, Helsinki, Finland

**Keywords:** Benign prostate hyperplasia, Transurethral resection of prostate, Laser vaporization, Open simple prostatectomy

## Abstract

**Purpose:**

To investigate postoperative mortality rates and risk factors for mortality after surgical treatment of benign prostate hyperplasia (BPH).

**Methods:**

All patients who underwent partial prostate excision/resection from 2004 to 2014 in Finland were retrospectively assessed for eligibility using a nationwide registry. Procedures were classified as transurethral resection of the prostate (TURP), laser vaporization of the prostate (laser), and open prostatectomy. Univariable and multivariable regression were used to analyze the association of age, Charlson comorbidity index (CCI), operation type, annual center operation volume, study era, atrial fibrillation, and prostate cancer diagnosis with 90 days postoperative mortality.

**Results:**

Among the 39,320 patients, TURP was the most common operation type for lower urinary tract symptoms in all age groups. The overall 90 days postoperative mortality was 1.10%. Excess mortality in the 90 days postoperative period was less than 0.5% in all age groups. Postoperative mortality after laser operations was 0.59% and 1.16% after TURP (*p* = 0.035). Older age, CCI score, and atrial fibrillation were identified as risk factors for postoperative mortality. Prostate cancer diagnosis and the center’s annual operation volume were not significantly associated with mortality. The most common underlying causes of death were malignancy (35.5%) and cardiac disease (30.9%).

**Conclusion:**

Elective urologic procedures for BPH are generally considered safe, but mortality increases with age. Laser operations may be associated with lower mortality rates than the gold standard TURP. Thus, operative risks and benefits must be carefully considered on a case-by-case basis. Further studies comparing operation types are needed.

**Supplementary Information:**

The online version contains supplementary material available at 10.1007/s00345-022-03999-0.

## Introduction

Lower urinary tract symptoms (LUTS) are increasingly common due to the aging population. Even though medical treatment for benign prostate hyperplasia (BPH) has improved markedly, surgical procedures are often still necessary.

Surgical treatment, especially endourology, is regarded as relatively safe; however, there seems to be a risk of serious complications associated with older age and comorbidities [1]. Hence, due to population aging, surgeons need to more carefully consider the risks and benefits of different procedures and take comorbidities into account [2]. While transurethral resection of the prostate (TURP) still remains the therapy of choice for benign prostatic obstruction, several transurethral ablative techniques have been developed. Of these less invasive techniques, transurethral laser surgery has become a permanent fixture alongside TURP. With the development of new techniques, open prostatectomy is still utilized (particularly for large prostates), but it is becoming less common [2–4].

Considering the safety of elderly and comorbid patients, it is relevant to ask whether urologists should choose laser ablation over traditional techniques to treat BPH. The estimate of mortality risk after endourologic surgery for BPH varies significantly, even among large-scale studies: 0.1–0.62% after TURP, 0.46–0.58% after laser vaporization of the prostate, and 0.35–0.51% after open prostatectomy [1, 2, 5]. Furthermore, there are no clear-cut data available that can be used to determine whether the postoperative mortality rates of these procedures are different [4].

Therefore, the purpose of this study was to examine the mortality related to procedures performed to treat BPH nationwide in Finland and to identify potential risk factors for increased mortality.

## Materials and methods

### Study design and patients

All patients undergoing a partial excision of prostate procedure classified in the Nordic Medico-Statistical Committee (NOMESCO) Classification of Surgical Procedures (NCSP) as open prostatectomy (KED00, KED10), transurethral resection of prostate (KED22, KED33, KED76) and as laser vaporization of the prostate (KED52) between 2004 and 2014 in Finland were identified from the Care Register for Healthcare and assessed for eligibility. Only the first procedure within the study period was included. The study population consisted of elective patients who came for the procedure from home; therefore, patients arriving from institutional care and patients with missing housing condition information or mortality data were excluded. Procedures administered as an emergency operation were also excluded. Patients diagnosed with a non-prostate malignancy or neoplasm of the urinary system were excluded. Detailed exclusions and study cohort selection flowdiagram are available in supplementary table 1 and supplementary figure 1.

### Data sources and permissions

This study was based on a nationwide administrative database of the Finnish Institute for Health and Welfare. The data were collected from the Care Register for Healthcare, the Official Statistics of Finnish causes of death register, and the Finnish Cancer Registry. The combined database included data on patients’ admission and discharge from inpatient care, day surgeries, ICD-10 diagnosis codes and procedural codes during admission, time and causes of death, and date and type of cancer diagnosis [6]. These registries are mandated by law and cover the entire Finnish population.

This study was approved by the National Institute for Health and Welfare of Finland (permission no.: THL/2245/5.05.00/2019) and Statistics Finland (TK-53-484-20). The legal basis for processing personal data was public interest and scientific research (EU General Data Protection Regulation 2016/679, Article 6(1)(e) and Article 9(2)(j); Data Protection Act, Sections 4 and 6). Due to the retrospective study design, informed consent was waived, and the participants were not contacted.

### Outcome definitions

The primary outcome of interest was postoperative death within 90 days. The secondary outcome was death within 1 year of the index operation. Each patient’s comorbidity burden was presented with the Charlson comorbidity index (CCI) calculated from the ICD-10 codes [7, 8]. As a surrogate for use of oral anticoagulation (OAC), atrial fibrillation was examined as an independent factor [6]. Operating centers were divided into three groups based on their annual surgical volume (<50, 50–100, and >100 operations annually). Cause of death was divided into the underlying cause of death (the disease or injury that initiated the chain of morbid events that led directly or inevitably to death) and the immediate cause of death (the final disease, injury, or complication that directly caused death). Underlying and immediate causes of death were categorized into eight groups based on clinical or anatomical criteria (the list of causes of death and categorizations are detailed in supplementary table 2).

### Statistical analyses

Differences between the study groups were evaluated using the *t* test and the chi-squared test. Outcomes were studied using a modified Poisson regression with robust error variances [9]. Variables in multivariable models were predetermined clinically. Excess postoperative mortality was calculated by subtracting the baseline all-cause mortality in the corresponding age-, sex-, and calendar year-specific groups in the total Finnish population from postoperative all-cause mortality [6]. Excess postoperative mortality calculations are available in supplementary table 3. Results are given as the mean, median, percentage, or relative risk (RR) with 95% confidence intervals (CIs). Statistical significance was inferred by a *p* value < 0.05. Analyses were performed with SAS version 9.4 (SAS Institute Inc., Cary, NC, USA).

## Results

A total of 45,134 procedures were performed on 41,168 patients in 60 operating centers between 2004 and 2014. After exclusions based on the aforementioned criteria, 39,320 patients were included in the study population, and their data were analyzed (Supplementary Figure 1). The characteristics of the study population are presented in Table 1. During the study period, 34,558 TURP, 3715 laser vaporization, and 1047 open prostatectomy procedures were performed. TURP was the most common operation type in all age groups. The majority of patients (70%) had a CCI score of 0. Only a fraction (7%) of the patients had atrial fibrillation (7%) or prostate cancer (9%). Most of the procedures were performed in centers with large annual operating volumes.

**Table 1:** Baseline features of the study population

Variable	All patients *N* (%)	Baseline features				
		Age group (years)				
		< 60	60–69	70–79	≥ 80	*p* value*
		*N* (%)	*N* (%)	*N* (%)	N (%)	
No of patients	39,320	4284 (10.9)	13,015 (33.1)	15,233 (38.7)	6788 (17.3)	
CCI score						
0	27,660 (70.4)	3669 (85.6)	10,159 (78.1)	10,181 (66.8)	3651 (53.8)	< 0.0001
1	4520 (11.5)	275 (6.4)	1238 (9.5)	1956 (12.8)	1051 (15.5)	
2	5200 (13.2)	268 (6.3)	1209 (9.3)	2273 (14.9)	1450 (21.4)	
3	1201 (3.1)	45 (1.1)	239 (1.8)	520 (3.4)	397 (5.9)	
≥ 4	739 (1.9)	27 (0.6)	170 (1.3)	303 (2.0)	239 (3.5)	
Atrial fibrillation	2732 (7.0)	67 (1.6)	575 (4.4)	1278 (8.4)	812 (12.0)	< 0.0001
Prostate cancer	4284 (9.2)	141 (3.3)	680 (5.2)	1575 (10.3)	1207 (17.8)	< 0.0001
Operation type						
TURP	34,558	3812 (89.0)	11,269 (86.6)	13,444 (88.3)	6033 (88.9)	< 0.0001
Laser	3715	402 (9.4)	1431 (11.0)	1297 (8.5)	585 (8.6)	
Open	1047	70 (1.6)	315 (2.4)	492 (3.2)	170 (2.5)	
Annual operation volume						
< 50	6561	774 (18.1)	2186 (16.8)	2524 (16.6)	1077 (15.9)	0.044
50–100	7977	851 (19.9)	2651 (20.4)	3137 (20.6)	1338 (19.7)	
> 100	24,782	2659 (62.1)	8178 (62.8)	9572 (62.8)	4373 (64.4)	

*indicating the value of p < 0.05

The association of factors with the 90 days postoperative mortality is presented in Table 2. Among the 39,320 patients who underwent a procedure for BPH, 431 died during the 90 days postoperative follow-up, yielding a 90 days postoperative mortality rate of 1.10%. Men who underwent TURP had a mortality rate of 1.16%; this was significantly higher than the 0.59% in those who underwent laser vaporization in both univariable (*p* = 0.002) and multivariable models (*p* = 0.035) (Table 2). The 90 days postoperative mortality after open prostatectomy was 0.67%. Also, increasing age, increasing CCI score, diagnosis of atrial fibrillation, and earlier study era were all independently associated with increasing mortality. Patients with prostate cancer had increased mortality (3.66%, *p* <0.0001) in the univariable model, but when taking other known variables into account in the multivariable model, diagnosis of prostate cancer per se did not increase mortality. In addition, the operational volume of the center was not independently associated with mortality.

**Table 2:** Univariable and multivariable analysis of 90 days postoperative mortality

Variable	Mortality (%)	90 days mortality			
		Univariable		Multivariable	
		RR (95% CI)	*p* value	RR (95% CI)	*p* value
Age-group			< 0.0001		< 0.0001
< 60	0.26	1 (reference)		1 (reference)	
60–69	0.47	1.83 (0.96–3.47)	0.066	1.59 (0.84–3.02)	0.154
70–79	1.13	4.40 (2.39–8.08)	< 0.0001	3.03 (1.64–5.60)	0.0004
≥ 80	2.75	10.73 (5.85–19.68)	< 0.0001	5.83 (3.13–10.88)	< 0.0001
CCI score			< 0.0001		< 0.0001
0	0.50	1 (reference)		1 (reference)	
1	1.44	2.90 (2.16–3.89)	< 0.0001	2.34 (1.73–3.17)	< 0.0001
2	2.79	5.63 (4.47–7.10)	< 0.0001	3.75 (2.81–4.99)	< 0.0001
3	4.08	8.24 (5.98–11.36)	< 0.0001	5.16 (3.51–7.57)	< 0.0001
≥ 4	4.74	9.56 (6.65–13.76)	< 0.0001	6.20 (4.15–9.27)	< 0.0001
Atrial fibrillation					
No	0.98	1 (reference)		1 (reference)	
Yes	2.60	2.64 (2.05–3.40)	< 0.0001	1.53 (1.18–1.99)	0.001
Prostate cancer					
No	0.84	1 (reference)		1 (reference)	
Yes	3.66	4.38 (3.58–5.36)	< 0.0001	1.20 (0.92–1.57)	0.170
Operation type			< 0.0001		0.029
TURP	1.16	1 (reference)		1 (reference)	
Laser	0.59	0.51 (0.33–0.78)	0.002	0.63 (0.41–0.97)	0.035
Open	0.67	0.57 (0.27–1.21)	0.145	0.72 (0.34–1.51)	0.382
Annual operation volume			0.288		0.586
< 50	0.93	0.83 (0.63–1.10)	0.200	0.88 (0.67–1.16)	0.367
50–100	1.18	1.06 (0.83–1.34)	0.635	1.03 (0.81–1.29)	0.834
> 100	1.11	1 (reference)		1 (reference)	
Study era					
2004–2009	1.20	1 (reference)		1 (reference)	
2010–2014	0.97	0.83 (0.60–1.13)	0.238	0.72 (0.59–0.88)	0.001

During the 90 days postoperative period, the most common underlying causes of death were malignancy (35.5%) and cardiac disease (30.9%). The 1 year postoperative mortality rate was 4.6%. Similar to the 90 days postoperative period, the dominant causes of death during the 1 year postoperative period were malignancy (45.8%) and cardiac disease (23.2%). When the baseline and postoperative mortality in different age groups were compared, it was found that the excess mortality increased with increasing age, and was <0.5% in the 90 days postoperative period and <2% in the 1 year postoperative period in all age groups. The causes of death are depicted in supplementary table 3, and baseline and BPH treatment-related excess mortality are available in supplementary table 4.

## Discussion

We studied postoperative mortality and mortality risk factors among Finnish men who underwent surgery for BPH in Finland. TURP was by far the most common operation type for BPH in Finland from 2004 to 2014. The overall mortality rate of the study population was 1.10% during the 90 days postoperative period, and the most common underlying causes of death were malignancy and cardiac disease. Laser vaporization had a lower mortality (0.59%) when compared to TURP (1.16%). Aging, CCI score, atrial fibrillation, and study period were identified as independent risk factors for higher postoperative mortality. The excess 90 days postoperative mortality rate was nevertheless low (<0.5%) in all age groups.

The results show that there is mortality related to elective endourologic procedures for BPH. Even though the excess mortality was low in our findings, it was significantly associated with older age. The 90 days postoperative mortality after TURP was 1.16% in our study, which is considerably higher than in a nationwide BPH treatment-related mortality recently reported by Eredics et al (0.5 %). This difference might be explained by the fact that Eredics et al. included only in-hospital mortality [10]. In our study, the mortality rate was based on the Causes of Death Registry, covering all deaths that occurred during the study period, irrespective of place of death. Another difference between the studies was the inclusion of patients with established prostate cancer (PCa). Including patients with prostate cancer in our study is justified, since the majority of these patients experience bladder outlet obstruction mostly because of concomitant BPH. Also, Crow et al. reported that there seems to be no excess postoperative mortality or complications after TURP in PCa patients [11]. More importantly, in our study, prostate cancer diagnosis was not found to be an independent risk factor for higher postoperative mortality.

This study is similar to Gilfrich and coworkers’ 2016 large study of 95,577 cases from a nationwide German health insurance database. The research design differed in that Gilfrich et al. studied 30 days postoperative mortality, whereas our study focused on 90 days postoperative mortality. Nevertheless, the postoperative mortalities were surprisingly similar after laser vaporization (0.58 %) and open prostatectomy (0.51 %), even when disregarding the time frame difference [2].

Bhojani et al. compared TURP and laser operations using data from the American College of Surgeons National Surgical Quality Improvement Program database (2006–2011). They found that laser vaporization of the prostate was associated with decreased blood transfusions, length of stay, and reintervention rates, but there was no significant difference in perioperative mortality between laser (0.3%) or TURP (0.4%). Advanced age and non-Caucasian race increased the risk of morbidity and mortality, whereas normal preoperative albumin and higher hematocrit levels were recognized as predictors of lower overall complications [4]. Patel et al. also used the National Surgical Quality Improvement Program database (2006–2011) to quantify complication rates, perioperative outcomes, and predictors for urological procedures. They found that TURP had the highest morbidity rate of prostatic endoscopic procedures (0.62 %). They also analyzed morbidity rates of photoselective vaporization of the prostate (GreenLight laser) (0.46 %), radical retropubic prostatectomy (0.35 %), and laparoscopic radical prostatectomy (0.11 %). Patel et al. studied the morbidity of urologic surgical procedures regardless of the indication for surgery. Therefore, it is safe to conclude that patients with PCa were included in this patient cohort. The difference in the 30 days postoperative mortality after TURP between the studies by Patel et al. and Bhojani et al. is 0.22%. This difference could hypothetically be explained by the different participant exclusion/inclusion criteria—Bhojani et al. did not report the possible exclusion of PCa patients [1]*.*

The hypothesis that laser operations for bladder outlet obstruction are lower-risk procedures and, therefore, are more frequently performed in elderly men than TURP or open prostatectomy is only partly supported by our results. Although there is a statistically significant association between age and operation type, the differences are small, and laser procedures are not more commonly performed in elderly men. More importantly, men who underwent laser procedures had significantly lower 90 days postoperative mortality compared to men who underwent TURP. However, no consensus prevails concerning the issue. Bhojani et al. found no difference in overall complications or perioperative mortality between TURP and laser operations [4], whereas Gilfrich et al. concluded that laser operations demonstrated favorable results for transfusions and bleeding, but increased long-term reinterventions when compared to TURP [2]. Even though postoperative mortality after a laser procedure was significantly lower than that after TURP in both the uni- and multivariable analyses in the current study, there might be unidentified variables and/or factors influencing these results.

There is an ongoing dialogue about whether oral anticoagulation should be ceased for TURP. Patients taking these medications have a higher rate of perioperative bleeding. However, if prescribed for secondary prevention, withholding OAC is associated with an increased rate of cardiovascular and cerebrovascular complications [12, 13]. Although we acknowledge that OAC is used in a variety of medical conditions and not solely for atrial fibrillation, in our study, atrial fibrillation was used as a surrogate for oral anticoagulation. Due to the retrospective nature of this study and the lack of detailed clinical information, it was not possible to identify patients who ceased OAC preoperatively. However atrial fibrillation was found to be an independent risk factor for increased mortality (2.60%). In the future, further studies on on-going OAC and the choice of operation type for LUTS are needed.

To study the real-world mortality data of patients with BPH who had undergone surgery, patients with PCa were included in the study cohort. PCa and BPH are not mutually exclusive, and even though the likelihood of detecting incidental PCa by surgery has decreased in the era of prostate-specific antigen (PSA) testing, 5.2–6.4% of newly identified PCas are still detected after surgery for BPH [14–16]. The inclusion of PCa patients may elevate the total mortality of the study population and mortality related to TURP, since postoperative 90 days mortality following palliative TURP is estimated to be 3.4% [17]. At the same time, patients who had been diagnosed with urinary system tumors were excluded from this study to examine actual KED procedures. This exclusion is justified, since when a physician is treating or diagnosing a urinary system neoplasm, they might plan to take a sample of the prostate, which may lead to entering a KED procedure code into the surgical report, even though the actual KED procedure was not performed.

This study was based on nationwide data from the Finnish Institute for Health and Welfare. The resulting data set of 39,320 patients over a 10 years period is a major strength of this study. In addition, the registry covers every hospital in Finland, and the data are truly nationwide. A general limitation of this study is the lack of detailed clinical information, and the data are limited to diagnosis and operation coding. Due to this deficiency, we were unable to identify and grade postoperative complications that may have caused death. Moreover, since the NCSP does not separate monopolar and bipolar TURP or specific types of laser operations, we were unable to classify operation types more accurately; therefore, certain differences within the operation groups may have remained unnoticed.

In conclusion, surgical treatment for LUTS seems to be safe for all age groups since the excess mortality after procedures was found to be less than 0.5%. In this nationwide cohort study, the results indicated a lower postoperative mortality after laser operations than after TURP. Aging, CCI score, and atrial fibrillation were identified as independent risk factors for higher postoperative mortality. Therefore, considering the risks and benefits of operating on a case-by-case basis is strongly recommended.

## Supplementary Information

Below is the link to the electronic supplementary material.

Supplementary file1 (DOCX 162 KB)
