# Supplementary material for: Mortality after surgery for benign prostate hyperplasia: a nationwide cohort study
Source: World J Urol. 2022 Apr 16;40(7):1785–91. doi: 10.1007/s00345-022-03999-0 (PMC9236974; doi:10.1007/s00345-022-03999-0)
Supplement: Supplementary file 1 — Supplementary file1 (DOCX 162 KB) [file 345_2022_3999_MOESM1_ESM.docx]

**Supplementary table 1.** Excluded ICD10 and NCSP codes

| **Exclusion based on ICD10 diagnoses (n=1719)** | | |
| --- | --- | --- |
|  |  |  |
| **ICD10** | **N** |  |
| C66 | 11 | Malignant neoplasm of ureter |
| C67.0 | 36 | Malignant neoplasm of trigone of bladder |
| C67.1 | 25 | Malignant neoplasm of dome of bladder |
| C67.2 | 168 | Malignant neoplasm of lateral wall of bladder |
| C67.3 | 5 | Malignant neoplasm of anterior wall of bladder |
| C67.4 | 57 | Malignant neoplasm of posterior wall of bladder |
| C67.5 | 3 | Malignant neoplasm of bladder neck |
| C67.6 | 1 | Malignant neoplasm of ureteric orifice |
| C67.7 | 1 | Malignant neoplasm of urachus |
| C67.8 | 557 | Malignant neoplasm of overlapping sites of bladder |
| C67.9 | 444 | Malignant neoplasm of bladder, unspecified |
| C68.0 | 13 | Malignant neoplasm of urethra |
| C68.1 | 1 | Malignant neoplasm of paraurethral glands |
| C68.8 | 5 | Malignant neoplasm of overlapping sites of urinary organs |
| C68.9 | 1 | Malignant neoplasm of urinary organ, unspecified |
| D30.2 | 1 | Benign neoplasm of ureter |
| D30.3 | 26 | Benign neoplasm of bladder |
| D30.4 | 3 | Benign neoplasm of urethra |
| D40.0 | 81 | Neoplasm of uncertain behavior of prostate |
| D41.2 | 1 | Neoplasm of uncertain behavior of ureter |
| D41.3 | 20 | Neoplasm of uncertain behavior of urethra |
| D41.4 | 251 | Neoplasm of uncertain behavior of bladder |
| D41.7 | 1 | Neoplasm of uncertain or unknown behaviour: Other urinary organs |
| D41.9 | 5 | Neoplasm of uncertain behavior of unspecified urinary organ |
|  |  |  |
| **Excluded NCSP operations** | | |
|  |  |  |
| **NCSP** | **N** |  |
| KED58 | 5 | Laser resection of prostate with ultrasound |
| KED62 | 70 | Transurethral needle ablation of prostate |
| KED72 | 71 | Transurethral microwave therapy of prostate |
| KED96 | 43 | Other partial excision of prostate |
| KED98 | 26 | Other transurethral partial excision of prostate |
| * |  |  |

*All combinations of multiple KED procedures during treatment period were excluded from analysis

**Supplementary figure 1.** Study cohort selection flowdiagram

**

**

**Supplementary table 2**. Cause of death statistics

|  |  |  |  |  |  |  |  |  |  |
| --- | --- | --- | --- | --- | --- | --- | --- | --- | --- |
|  |  | **90-day mortality** | | | | |  | **1-year mortality** | |
|  |  | **Underlying cause of death** | |  | **Immediate cause(s) of death** | |  | **Underlying cause of death** | |
| **Cause of death** | | **N** | **ICD10** |  | **N** | **ICD10** |  | **N** | **ICD10** |
| **Cardiac** | |  |  |  |  |  |  |  |  |
|  | **Ischemic** | 3 | I210 |  | 1 | I209 |  | 16 | I210 |
|  |  | 4 | I211 |  | 4 | I210 |  | 9 | I211 |
|  |  | 7 | I212 |  | 3 | I211 |  | 21 | I212 |
|  |  | 1 | I213 |  | 7 | I212 |  | 3 | I213 |
|  |  | 6 | I214 |  | 1 | I213 |  | 29 | I214 |
|  |  | 32 | I219 |  | 5 | I214 |  | 89 | I219 |
|  |  | 2 | I228 |  | 24 | I219 |  | 5 | I228 |
|  |  | 1 | I229 |  | 3 | I228 |  | 7 | I229 |
|  |  | 47 | I251 |  | 2 | I229 |  | 3 | I250 |
|  |  | 4 | I255 |  | 1 | I250 |  | 157 | I251 |
|  |  | 2 | I258 |  | 46 | I251 |  | 3 | I252 |
|  |  | 7 | I259 |  | 5 | I252 |  | 5 | I255 |
|  |  |  |  |  | 2 | I258 |  | 5 | I258 |
|  |  |  |  |  | 5 | I259 |  | 18 | I259 |
|  |  |  |  |  |  |  |  |  |  |
|  | **Non-ischemic** | 5 | I350 |  | 1 | I080 |  | 1 | I080 |
|  |  | 3 | I352 |  | 30 | I10 |  | 2 | I340 |
|  |  | 2 | I422 |  | 2 | I340 |  | 1 | I342 |
|  |  | 3 | I48 |  | 1 | I342 |  | 10 | I350 |
|  |  | 1 | I500 |  | 5 | I350 |  | 1 | I351 |
|  |  | 2 | I509 |  | 3 | I351 |  | 3 | I352 |
|  |  | 1 | I515 |  | 1 | I352 |  | 7 | I420 |
|  |  |  |  |  | 1 | I359 |  | 3 | I422 |
|  |  |  |  |  | 1 | I420 |  | 1 | I426 |
|  |  |  |  |  | 1 | I442 |  | 9 | I48 |
|  |  |  |  |  | 2 | I461 |  | 3 | I500 |
|  |  |  |  |  | 2 | I469 |  | 1 | I501 |
|  |  |  |  |  | 30 | I48 |  | 6 | I509 |
|  |  |  |  |  | 2 | I495 |  | 2 | I515 |
|  |  |  |  |  | 2 | I499 |  | 2 | I517 |
|  |  |  |  |  | 10 | I500 |  |  |  |
|  |  |  |  |  | 9 | I501 |  |  |  |
|  |  |  |  |  | 20 | I509 |  |  |  |
|  |  |  |  |  | 1 | I517 |  |  |  |
|  |  |  |  |  | 1 | R570 |  |  |  |
|  |  |  |  |  |  |  |  |  |  |
| **Respiratory** | |  |  |  |  |  |  |  |  |
|  |  | 1 | J439 |  | 1 | J330 |  | 3 | J439 |
|  |  | 4 | J448 |  | 1 | J398 |  | 2 | J441 |
|  |  | 1 | J449 |  | 2 | J439 |  | 15 | J448 |
|  |  | 1 | J459 |  | 1 | J441 |  | 1 | J449 |
|  |  | 2 | J841 |  | 18 | J448 |  | 1 | J459 |
|  |  | 1 | J920 |  | 6 | J449 |  | 1 | J46 |
|  |  | 1 | J931 |  | 1 | J450 |  | 1 | J47 |
|  |  |  |  |  | 1 | J451 |  | 6 | J841 |
|  |  |  |  |  | 4 | J459 |  | 1 | J920 |
|  |  |  |  |  | 1 | J61 |  | 1 | J931 |
|  |  |  |  |  | 1 | J81 |  |  |  |
|  |  |  |  |  | 2 | J841 |  |  |  |
|  |  |  |  |  | 1 | J920 |  |  |  |
|  |  |  |  |  | 1 | J960 |  |  |  |
|  |  |  |  |  | 2 | J961 |  |  |  |
|  |  |  |  |  |  |  |  |  |  |
| **Genitourinary** | |  |  |  |  |  |  |  |  |
|  |  | 1 | N032 |  | 18 | N10 |  | 1 | N031 |
|  |  | 2 | N10 |  | 1 | N12 |  | 1 | N032 |
|  |  | 1 | N138 |  | 1 | N130 |  | 5 | N10 |
|  |  | 2 | N189 |  | 3 | N133 |  | 1 | N133 |
|  |  | 1 | N210 |  | 2 | N170 |  | 1 | N138 |
|  |  | 1 | N328 |  | 1 | N178 |  | 3 | N189 |
|  |  | 11 | N40 |  | 8 | N179 |  | 1 | N210 |
|  |  |  |  |  | 1 | N188 |  | 1 | N328 |
|  |  |  |  |  | 8 | N189 |  | 12 | N40 |
|  |  |  |  |  | 9 | N19 |  |  |  |
|  |  |  |  |  | 1 | N200 |  |  |  |
|  |  |  |  |  | 3 | N210 |  |  |  |
|  |  |  |  |  | 1 | N320 |  |  |  |
|  |  |  |  |  | 23 | N40 |  |  |  |
|  |  |  |  |  | 1 | N990 |  |  |  |
|  |  |  |  |  | 2 | R31 |  |  |  |
|  |  |  |  |  | 1 | R33 |  |  |  |
|  |  |  |  |  |  |  |  |  |  |
| **Vascular** | |  |  |  |  |  |  |  |  |
|  | **Stroke** | 1 | I601 |  | 1 | I613 |  | 2 | I601 |
|  |  | 1 | I609 |  | 2 | I633 |  | 1 | I602 |
|  |  | 3 | I610 |  | 2 | I634 |  | 2 | I609 |
|  |  | 1 | I611 |  | 2 | I639 |  | 15 | I610 |
|  |  | 1 | I619 |  | 2 | I691 |  | 3 | I611 |
|  |  | 1 | I630 |  | 16 | I693 |  | 2 | I613 |
|  |  | 5 | I633 |  |  |  |  | 1 | I614 |
|  |  | 2 | I634 |  |  |  |  | 1 | I616 |
|  |  | 1 | I635 |  |  |  |  | 1 | I618 |
|  |  | 6 | I639 |  |  |  |  | 6 | I619 |
|  |  | 5 | I693 |  |  |  |  | 3 | I630 |
|  |  |  |  |  |  |  |  | 1 | I631 |
|  |  |  |  |  |  |  |  | 1 | I632 |
|  |  |  |  |  |  |  |  | 13 | I633 |
|  |  |  |  |  |  |  |  | 5 | I634 |
|  |  |  |  |  |  |  |  | 2 | I635 |
|  |  |  |  |  |  |  |  | 46 | I639 |
|  |  |  |  |  |  |  |  | 1 | I64 |
|  |  |  |  |  |  |  |  | 2 | I691 |
|  |  |  |  |  |  |  |  | 19 | I693 |
|  |  |  |  |  |  |  |  |  |  |
|  | **Other** | 1 | I120 |  | 1 | D683 |  | 13 | I110 |
|  |  | 1 | I701 |  | 1 | I110 |  | 2 | I119 |
|  |  | 1 | I709 |  | 3 | I119 |  | 2 | I120 |
|  |  | 3 | I710 |  | 1 | I260 |  | 2 | I132 |
|  |  | 3 | I713 |  | 9 | I269 |  | 1 | I260 |
|  |  | 1 | I714 |  | 2 | I312 |  | 1 | I269 |
|  |  | 1 | I719 |  | 1 | I652 |  | 1 | I671 |
|  |  | 2 | I802 |  | 2 | I672 |  | 2 | I672 |
|  |  |  |  |  | 1 | I676 |  | 1 | I701 |
|  |  |  |  |  | 1 | I679 |  | 4 | I702 |
|  |  |  |  |  | 1 | I700 |  | 2 | I709 |
|  |  |  |  |  | 3 | I702 |  | 4 | I710 |
|  |  |  |  |  | 5 | I709 |  | 1 | I711 |
|  |  |  |  |  | 1 | I710 |  | 5 | I713 |
|  |  |  |  |  | 1 | I712 |  | 1 | I714 |
|  |  |  |  |  | 1 | I802 |  | 2 | I718 |
|  |  |  |  |  |  |  |  | 1 | I719 |
|  |  |  |  |  |  |  |  | 1 | I743 |
|  |  |  |  |  |  |  |  | 1 | I801 |
|  |  |  |  |  |  |  |  | 4 | I802 |
|  |  |  |  |  |  |  |  |  |  |
| **Gastrointestinal** | | |  |  |  |  |  |  |  |
|  |  | 1 | K254 |  | 1 | K250 |  | 1 | K20 |
|  |  | 1 | K260 |  | 1 | K260 |  | 3 | K254 |
|  |  | 1 | K403 |  | 3 | K528 |  | 1 | K255 |
|  |  | 1 | K52.80 |  | 1 | K550 |  | 2 | K260 |
|  |  | 1 | K559 |  | 1 | K551 |  | 1 | K261 |
|  |  | 1 | K560 |  | 1 | K567 |  | 1 | K264 |
|  |  | 2 | K562 |  | 1 | K572 |  | 1 | K359 |
|  |  | 1 | K565 |  | 2 | K631 |  | 1 | K403 |
|  |  | 2 | K593 |  | 4 | K650 |  | 1 | K441 |
|  |  | 1 | K631 |  | 1 | K659 |  | 1 | K518 |
|  |  | 2 | K700 |  | 1 | K710 |  | 1 | K528 |
|  |  | 1 | K703 |  | 2 | K746 |  | 1 | K550 |
|  |  | 1 | K746 |  | 1 | K819 |  | 1 | K559 |
|  |  | 1 | K831 |  | 1 | K913 |  | 2 | K560 |
|  |  | 1 | K860 |  | 1 | K922 |  | 3 | K562 |
|  |  |  |  |  |  |  |  | 2 | K565 |
|  |  |  |  |  |  |  |  | 1 | K567 |
|  |  |  |  |  |  |  |  | 1 | K572 |
|  |  |  |  |  |  |  |  | 3 | K593 |
|  |  |  |  |  |  |  |  | 1 | K631 |
|  |  |  |  |  |  |  |  | 4 | K700 |
|  |  |  |  |  |  |  |  | 3 | K703 |
|  |  |  |  |  |  |  |  | 1 | K711 |
|  |  |  |  |  |  |  |  | 4 | K746 |
|  |  |  |  |  |  |  |  | 1 | K800 |
|  |  |  |  |  |  |  |  | 1 | K831 |
|  |  |  |  |  |  |  |  | 1 | K859 |
|  |  |  |  |  |  |  |  | 1 | K860 |
|  |  |  |  |  |  |  |  |  |  |
| **Infection** | |  |  |  |  |  |  |  |  |
|  |  | 1 | A09 |  | 2 | A047 |  | 1 | A047 |
|  |  | 1 | A415 |  | 2 | A09 |  | 1 | A09 |
|  |  | 1 | G002 |  | 1 | A401 |  | 1 | A392 |
|  |  | 1 | J159 |  | 1 | A403 |  | 1 | A403 |
|  |  | 2 | J189 |  | 2 | A410 |  | 3 | A410 |
|  |  | 6 | J440 |  | 1 | A414 |  | 2 | A411 |
|  |  |  |  |  | 3 | A415 |  | 1 | A415 |
|  |  |  |  |  | 2 | A418 |  | 1 | A418 |
|  |  |  |  |  | 6 | A419 |  | 1 | A46 |
|  |  |  |  |  | 1 | A46 |  | 2 | G002 |
|  |  |  |  |  | 1 | I330 |  | 1 | I309 |
|  |  |  |  |  | 1 | J13 |  | 1 | I38 |
|  |  |  |  |  | 13 | J159 |  | 1 | J13 |
|  |  |  |  |  | 11 | J180 |  | 8 | J159 |
|  |  |  |  |  | 1 | J181 |  | 1 | J180 |
|  |  |  |  |  | 43 | J189 |  | 5 | J189 |
|  |  |  |  |  | 1 | J440 |  | 26 | J440 |
|  |  |  |  |  | 9 | J690 |  | 1 | J851 |
|  |  |  |  |  | 1 | J851 |  | 5 | N390 |
|  |  |  |  |  | 2 | N300 |  | 1 | N459 |
|  |  |  |  |  | 1 | N309 |  |  |  |
|  |  |  |  |  | 1 | N390 |  |  |  |
|  |  |  |  |  |  |  |  |  |  |
|  |  |  |  |  |  |  |  |  |  |
| **Malignacy** | |  |  |  |  |  |  |  |  |
|  |  | 1 | C131 |  | 1 | C150 |  | 1 | C102 |
|  |  | 1 | C155 |  | 1 | C163 |  | 1 | C119 |
|  |  | 1 | C159 |  | 1 | C185 |  | 1 | C131 |
|  |  | 2 | C160 |  | 1 | C20 |  | 2 | C151 |
|  |  | 1 | C182 |  | 1 | C220 |  | 1 | C153 |
|  |  | 1 | C185 |  | 1 | C341 |  | 2 | C155 |
|  |  | 2 | C187 |  | 1 | C443 |  | 1 | C158 |
|  |  | 4 | C20 |  | 36 | C61 |  | 1 | C159 |
|  |  | 1 | C220 |  | 1 | C64 |  | 4 | C160 |
|  |  | 1 | C250 |  | 1 | C671 |  | 2 | C162 |
|  |  | 1 | C259 |  | 1 | C679 |  | 4 | C163 |
|  |  | 1 | C269 |  | 1 | C689 |  | 1 | C166 |
|  |  | 1 | C340 |  | 1 | C900 |  | 3 | C168 |
|  |  | 4 | C341 |  |  |  |  | 2 | C169 |
|  |  | 1 | C342 |  |  |  |  | 1 | C171 |
|  |  | 2 | C343 |  |  |  |  | 1 | C179 |
|  |  | 3 | C348 |  |  |  |  | 2 | C180 |
|  |  | 2 | C349 |  |  |  |  | 1 | C181 |
|  |  | 1 | C459 |  |  |  |  | 5 | C182 |
|  |  | 100 | C61 |  |  |  |  | 3 | C185 |
|  |  | 5 | C64 |  |  |  |  | 8 | C187 |
|  |  | 1 | C670 |  |  |  |  | 2 | C188 |
|  |  | 1 | C674 |  |  |  |  | 1 | C19 |
|  |  | 1 | C678 |  |  |  |  | 19 | C20 |
|  |  | 1 | C679 |  |  |  |  | 9 | C220 |
|  |  | 1 | C689 |  |  |  |  | 5 | C221 |
|  |  | 2 | C80 |  |  |  |  | 1 | C229 |
|  |  | 1 | C821 |  |  |  |  | 1 | C249 |
|  |  | 1 | C830 |  |  |  |  | 15 | C250 |
|  |  | 1 | C833 |  |  |  |  | 1 | C251 |
|  |  | 1 | C839 |  |  |  |  | 2 | C258 |
|  |  | 3 | C900 |  |  |  |  | 7 | C259 |
|  |  | 2 | C911 |  |  |  |  | 3 | C269 |
|  |  | 1 | C927 |  |  |  |  | 1 | C320 |
|  |  |  |  |  |  |  |  | 1 | C322 |
|  |  |  |  |  |  |  |  | 5 | C340 |
|  |  |  |  |  |  |  |  | 26 | C341 |
|  |  |  |  |  |  |  |  | 3 | C342 |
|  |  |  |  |  |  |  |  | 15 | C343 |
|  |  |  |  |  |  |  |  | 12 | C348 |
|  |  |  |  |  |  |  |  | 14 | C349 |
|  |  |  |  |  |  |  |  | 1 | C382 |
|  |  |  |  |  |  |  |  | 1 | C434 |
|  |  |  |  |  |  |  |  | 3 | C435 |
|  |  |  |  |  |  |  |  | 1 | C436 |
|  |  |  |  |  |  |  |  | 1 | C437 |
|  |  |  |  |  |  |  |  | 1 | C438 |
|  |  |  |  |  |  |  |  | 1 | C439 |
|  |  |  |  |  |  |  |  | 1 | C443 |
|  |  |  |  |  |  |  |  | 5 | C450 |
|  |  |  |  |  |  |  |  | 1 | C451 |
|  |  |  |  |  |  |  |  | 1 | C459 |
|  |  |  |  |  |  |  |  | 1 | C494 |
|  |  |  |  |  |  |  |  | 1 | C495 |
|  |  |  |  |  |  |  |  | 1 | C509 |
|  |  |  |  |  |  |  |  | 532 | C61 |
|  |  |  |  |  |  |  |  | 20 | C64 |
|  |  |  |  |  |  |  |  | 2 | C65 |
|  |  |  |  |  |  |  |  | 1 | C670 |
|  |  |  |  |  |  |  |  | 1 | C671 |
|  |  |  |  |  |  |  |  | 1 | C672 |
|  |  |  |  |  |  |  |  | 1 | C674 |
|  |  |  |  |  |  |  |  | 10 | C678 |
|  |  |  |  |  |  |  |  | 8 | C679 |
|  |  |  |  |  |  |  |  | 1 | C689 |
|  |  |  |  |  |  |  |  | 2 | C711 |
|  |  |  |  |  |  |  |  | 3 | C73 |
|  |  |  |  |  |  |  |  | 8 | C80 |
|  |  |  |  |  |  |  |  | 1 | C821 |
|  |  |  |  |  |  |  |  | 2 | C830 |
|  |  |  |  |  |  |  |  | 5 | C833 |
|  |  |  |  |  |  |  |  | 1 | C838 |
|  |  |  |  |  |  |  |  | 2 | C839 |
|  |  |  |  |  |  |  |  | 1 | C845 |
|  |  |  |  |  |  |  |  | 8 | C900 |
|  |  |  |  |  |  |  |  | 1 | C901 |
|  |  |  |  |  |  |  |  | 6 | C911 |
|  |  |  |  |  |  |  |  | 4 | C920 |
|  |  |  |  |  |  |  |  | 1 | C921 |
|  |  |  |  |  |  |  |  | 1 | C924 |
|  |  |  |  |  |  |  |  | 1 | C927 |
|  |  |  |  |  |  |  |  |  |  |
| **Miscellaneous** | |  |  |  |  |  |  |  |  |
|  |  | 1 | B181 |  | 1 | B378 |  | 1 | B181 |
|  |  | 1 | B909 |  | 1 | B941 |  | 2 | B909 |
|  |  | 1 | E102 |  | 1 | B99 |  | 1 | D120 |
|  |  | 2 | E112 |  | 1 | D381 |  | 1 | D379 |
|  |  | 1 | E116 |  | 1 | D467 |  | 1 | D381 |
|  |  | 1 | E117 |  | 1 | D481 |  | 1 | D45 |
|  |  | 1 | F019 |  | 1 | D649 |  | 3 | D471 |
|  |  | 1 | G122 |  | 1 | D869 |  | 1 | D473 |
|  |  | 3 | G20 |  | 1 | E040 |  | 1 | D479 |
|  |  | 8 | G301 |  | 1 | E052 |  | 1 | D868 |
|  |  | 1 | G309 |  | 1 | E108 |  | 1 | D869 |
|  |  | 1 | G312 |  | 1 | E109 |  | 1 | E102 |
|  |  | 1 | G409 |  | 1 | E110 |  | 4 | E112 |
|  |  | 1 | L959 |  | 1 | E112 |  | 2 | E115 |
|  |  | 1 | V485 |  | 1 | E113 |  | 1 | E116 |
|  |  | 3 | W01 |  | 5 | E117 |  | 7 | E117 |
|  |  | 1 | W10 |  | 4 | E118 |  | 1 | E669 |
|  |  | 2 | W18 |  | 32 | E119 |  | 1 | E858 |
|  |  | 1 | W19 |  | 1 | E249 |  | 1 | F011 |
|  |  | 1 | W79 |  | 3 | E669 |  | 2 | F012 |
|  |  | 1 | X41 |  | 2 | E780 |  | 1 | F013 |
|  |  | 1 | X61 |  | 1 | F019 |  | 4 | F019 |
|  |  | 3 | X70 |  | 2 | F03 |  | 5 | F03 |
|  |  | 1 | X71 |  | 1 | F059 |  | 1 | F102 |
|  |  | 2 | X72 |  | 4 | F100 |  | 2 | F107 |
|  |  | 3 | X73 |  | 3 | F102 |  | 1 | F509 |
|  |  | 1 | X80 |  | 1 | F190 |  | 1 | G121 |
|  |  |  |  |  | 1 | F203 |  | 6 | G122 |
|  |  |  |  |  | 1 | F209 |  | 15 | G20 |
|  |  |  |  |  | 2 | F329 |  | 3 | G231 |
|  |  |  |  |  | 1 | F339 |  | 28 | G301 |
|  |  |  |  |  | 1 | F419 |  | 5 | G308 |
|  |  |  |  |  | 1 | F700 |  | 7 | G309 |
|  |  |  |  |  | 5 | G20 |  | 1 | G310 |
|  |  |  |  |  | 1 | G219 |  | 1 | G312 |
|  |  |  |  |  | 3 | G301 |  | 1 | G318 |
|  |  |  |  |  | 2 | G308 |  | 1 | G35 |
|  |  |  |  |  | 7 | G309 |  | 1 | G409 |
|  |  |  |  |  | 1 | G318 |  | 1 | G419 |
|  |  |  |  |  | 2 | G409 |  | 1 | G834 |
|  |  |  |  |  | 1 | G410 |  | 1 | G903 |
|  |  |  |  |  | 1 | G729 |  | 1 | L959 |
|  |  |  |  |  | 1 | G931 |  | 1 | M068 |
|  |  |  |  |  | 2 | J80 |  | 1 | M069 |
|  |  |  |  |  | 1 | L89 |  | 1 | M349 |
|  |  |  |  |  | 1 | L97 |  | 1 | M480 |
|  |  |  |  |  | 1 | M058 |  | 1 | Q446 |
|  |  |  |  |  | 1 | M244 |  | 1 | R999 |
|  |  |  |  |  | 1 | M316 |  | 1 | V031 |
|  |  |  |  |  | 1 | M45 |  | 1 | V041 |
|  |  |  |  |  | 3 | W01 |  | 1 | V284 |
|  |  |  |  |  | 1 | W06 |  | 1 | V485 |
|  |  |  |  |  | 1 | W10 |  | 1 | V847 |
|  |  |  |  |  | 3 | W18 |  | 21 | W01 |
|  |  |  |  |  | 1 | X41 |  | 1 | W05 |
|  |  |  |  |  | 1 | X59 |  | 1 | W06 |
|  |  |  |  |  | 1 | X70 |  | 2 | W07 |
|  |  |  |  |  | 1 | Y608 |  | 4 | W10 |
|  |  |  |  |  | 2 | Y831 |  | 2 | W13 |
|  |  |  |  |  | 1 | Y836 |  | 6 | W18 |
|  |  |  |  |  | 3 | Y838 |  | 3 | W19 |
|  |  |  |  |  | 1 | Y848 |  | 1 | W71 |
|  |  |  |  |  | 1 | Y850 |  | 1 | W79 |
|  |  |  |  |  |  |  |  | 1 | W92 |
|  |  |  |  |  |  |  |  | 2 | X00 |
|  |  |  |  |  |  |  |  | 3 | X41 |
|  |  |  |  |  |  |  |  | 5 | X45 |
|  |  |  |  |  |  |  |  | 2 | X59 |
|  |  |  |  |  |  |  |  | 3 | X61 |
|  |  |  |  |  |  |  |  | 2 | X62 |
|  |  |  |  |  |  |  |  | 2 | X64 |
|  |  |  |  |  |  |  |  | 9 | X70 |
|  |  |  |  |  |  |  |  | 1 | X71 |
|  |  |  |  |  |  |  |  | 3 | X72 |
|  |  |  |  |  |  |  |  | 8 | X73 |
|  |  |  |  |  |  |  |  | 1 | X80 |
|  |  |  |  |  |  |  |  | 1 | Y21 |
|  |  |  |  |  |  |  |  | 2 | Y86 |

**Supplementary table 3.** Causes of death

|  |  | **90-day mortality** | | |  | **One-year mortality** |
| --- | --- | --- | --- | --- | --- | --- |
|  |  |  | | |  |  |
| **Cause of death** | | **Underlying cause of death** |  | **Immediate cause(s) of death** |  | **Underlying cause of death** |
|  |  | **N (%)** |  | **N (%)** |  | **N (%)** |
| **Cardiac** | |  |  |  |  |  |
|  | Ischemic | 116 (26.9) |  | 109 (15.0) |  | 370 (20.3) |
|  | Non-ischemic | 17 (3.9) |  | 125 (17.1) |  | 52 (2.9) |
|  | Total | 133 (30.9) |  |  |  | 422 (23.2) |
|  |  |  |  |  |  |  |
| **Respiratory** | | 11 (2.6) |  | 43 (5.9) |  | 32 (1.8) |
|  |  |  |  |  |  |  |
| **Genitourinary** | | 19 (4.4) |  | 84 (11.5) |  | 26 (1.4) |
|  |  |  |  |  |  |  |
| **Vascular** | |  |  |  |  |  |
|  | Stroke | 27 (6.3) |  | 25 (3.4) |  | 127 (7.0) |
|  | Other | 13 (3.0) |  | 34 (4.7) |  | 51 (2.8) |
|  | Total | 40 (9.3) |  |  |  | 178 (9.8) |
|  |  |  |  |  |  |  |
| **Gastrointestinal** | | 18 (4.2) |  | 22 (3.0) |  | 45 (2.5) |
|  |  |  |  |  |  |  |
| **Infection** | | 12 (2.8) |  | 106 (14.5) |  | 64 (3.5) |
|  |  |  |  |  |  |  |
| **Malignancy** | | 153 (35.5) |  | 48 (6.6) |  | 834 (45.8) |
|  |  |  |  |  |  |  |
| **Miscellaneous** | | 45 (10.4) |  | 133 (18.2) |  | 220 (12.1) |
|  |  |  |  |  |  |  |
| **Total** | | **431** |  | **729** |  | **1,821** |

**Supplementary table 4.** Baseline and excess mortality

|  | **90-day mortality (%)** | | |  | **One-year mortality (%)** | | |
| --- | --- | --- | --- | --- | --- | --- | --- |
| **Age group (years)** | **Baseline** | **Postoperative** | **Excess** |  | **Baseline** | **Postoperative** | **Excess** |
| **<60** | 0.20 | 0.26 | 0.06 |  | 0.8 | 1.12 | 0.32 |
| **60–69** | 0.40 | 0.47 | 0.07 |  | 1.63 | 2.01 | 0.38 |
| **70–79** | 0.92 | 1.13 | 0.21 |  | 3.73 | 5.01 | 1.28 |
| **≥80** | 2.29 | 2.75 | 0.46 |  | 9.29 | 11.03 | 1.74 |

* Excess postoperative mortality was calculated by subtracting the baseline all-cause mortality in the corresponding age-, sex-, and calendar year-specific groups in the total Finnish population from postoperative all-cause mortality. Mortality was weighted within the age-groups according to the age distribution of the study population.
